# Pluronic 123 Liquid Lyotropic Crystals for Transdermal Delivery of Caffeic Acid—Insights from Structural Studies and Drug Release

**DOI:** 10.3390/gels10030181

**Published:** 2024-03-06

**Authors:** Martina Romeo, Elisabetta Mazzotta, Francesca Lovati, Michele Porto, Cesare Oliviero Rossi, Rita Muzzalupo

**Affiliations:** 1 Department of Pharmacy Health and Nutritional Sciences, University of Calabria, Via P. Bucci, 87036 Arcavacata di Rende, Italy; martina.romeo@unical.it (M.R.); mazzotta-elisabetta@libero.it (E.M.); francescalovati30@gmail.com (F.L.); 2 Department of Chemistry and Chemical Technologies, Cubo 14/D, University of Calabria, Via P. Bucci, 87036 Arcavacata di Rende, Italy; michele.porto@unical.it (M.P.);

**Keywords:** lyotropic liquid crystals, pluronic P123, gel-like phases, caffeic acid, transdermal drug delivery

## Abstract

Background: This study aims to evaluate the percutaneous permeation profiles of caffeic acid (CA) from the cubic and hexagonal liquid crystalline phases of Pluronic P123/water mixtures. Method: The resulting drug-loaded mesophases were subjected to characterisation through deuterium nuclear magnetic resonance spectroscopy and polarised optical microscopy observations. These analyses aimed to evaluate the structural changes that occurred in the mesophases loading with CA. Additionally, steady and dynamic rheology studies were conducted to further explore their mechanical properties and correlate them to the supramolecular structure. Finally, CA release experiments were carried out at two different temperatures to examine the behaviour of the structured systems in a physiological or hyperthermic state. Results: As the concentration of the polymer increases, an increase in the viscosity of the gel is noted; however, the addition of caffeic acid increases microstructure fluidity. It is observed that the temperature effect conforms to expectations. The increase in temperature causes a decrease in viscosity and, consequently, an increase in the rate of permeation of caffeic acid. Conclusions: The CA permeation profile from the prepared formulations is mostly dependent on the structural organisation and temperature. Cubic mesophase LLC 30/CA showed greater skin permeation with good accumulation in the skin at both tested temperatures.

## 1. Introduction

In recent years, antioxidants have gained increasing interest in the pharmaceutical, cosmetic, medical, and food sectors due to their many benefits. These prophylactic molecules play a crucial role in the prevention and management of pathologies associated with oxidative stress, such as cancer [1], atherosclerosis [2], diabetes [3], ocular diseases [4], and Alzheimer’s disease [5]. Their action involves inhibiting or reducing the effects caused by free radicals and oxidising compounds. Due to regulatory restrictions on the use of synthetic antioxidants, driven by potential risks associated with in vivo administration, there is a growing interest in natural antioxidants [6]. Among these, phenolic antioxidants act as scavengers of reactive species, including free radicals, actively participating in metal chelation during the oxidative process. The donation of an electron or hydrogen atoms stabilises free radicals, imparting antioxidant activity [7]. An example of a phenolic antioxidant is caffeic acid (CA), also known as 3,4-dihydroxycinnamic acid, found in blueberries, apples, cider, coffee, and propolis. In addition to its antioxidant properties, CA exhibits antimicrobial activity, anti-inflammatory activity, is known as a cancer inhibitor, and contributes to the prevention of heart diseases and atherosclerosis. Several studies have indeed demonstrated the ability of CA to counteract inflammation evident in various diseases, such as skin conditions like atopic dermatitis [8,9], and intestinal disorders like ulcerative colitis and Crohn’s disease [10]. CA has gained considerable attention in the pharmaceutical field as a promising photoprotective agent and for skincare. However, the poor water solubility and low chemical stability of CA require the use of suitable delivery systems to ensure solubilisation, protection from degradation, and pharmaceutical efficacy [11,12]. The field of nanotechnology has indeed focused extensively on antioxidant molecules due to their numerous benefits, despite their low bioavailability and improper release into undesired cellular compartments. Consequently, this has led to the development of antioxidant nanosystems [13,14] aimed at enhancing the effectiveness of conventional antioxidants, improving stability, increasing bioavailability, and ensuring a controlled and targeted release. In recent years, research on transdermal delivery systems has undergone considerable development, focusing on a wide range of drug carriers, such as liposomes, niosomes, microemulsions, transdermal patches, microneedles, etc. The first approved transdermal delivery system was the scopolamine patch for motion sickness in 1979. Since then, drugs, such as non-steroidal anti-inflammatory [15], antimicrobials [16], antioxidants [17] and anti-cancer [18], have been successfully formulated as transdermal drug delivery systems (TDDS). Transdermal drug delivery systems, such as lyotropic liquid crystals (LLC), are considered promising alternatives to improve pharmacological effectiveness, safety, and patient compliance [19]. The LLC are soft materials that combine their anisotropic order with the mechanical stability of a gel. A significant advantage of LLCs is their ability to act as a barrier that controls the rate of release of encapsulated drugs [20,21]. This allows a prolonged drug release while maintaining effective concentrations in the desired region. Lyotropic liquid crystals (LLC) or self-assembling materials are typically formed from water and surfactants in well-defined ratios. The structure-forming surfactants can absorb a certain amount of water and then spontaneously form gel-like phases with unique internal structures. They self-assemble into cubic, hexagonal (H_2_), and lamellar (Lα) mesophases, depending on the concentration of constituents and temperature [22]. Among the surfactants most used for LLC formulation are Pluronic surfactants, which are block copolymers consisting of repeating units of ethylene oxide (EO) and propylene oxide (PO), represented by the general formula (PEO)_n_(PPO)_m_(PEO)_n_. Pluronic P123 (EO_20_PO_70_EO_20_), depending on temperature and concentration, is capable of making various structures in water [23,24], as observed in the phase diagram of the P123-water system determined by Wanka et al. [25].

The purpose of this article is to evaluate the percutaneous permeation profile of CA from cubic and hexagonal liquid crystalline phases of Pluronic P123/water mixtures and to identify how transdermal drug delivery was affected by the gel-like microstructure at different bodily functions. In detail, permeation and diffusion studies were performed at 32° and 40 °C, to highlight any differences between physiological conditions while the data on the characterisation were carried out also at 25 °C to point out our formulation’s storage temperature. Mesophases obtained, with or without CA, were analysed using deuterium nuclear magnetic resonance spectroscopy (^2^H-NMR), polarised optical microscopy (POM) observations and via dynamic rheology experiments, to identify the influence of CA in the mesophases structures.

## 2. Results and Discussion

### 2.1. LLC Physical-Chemical Characterisation

**Figure 1:**
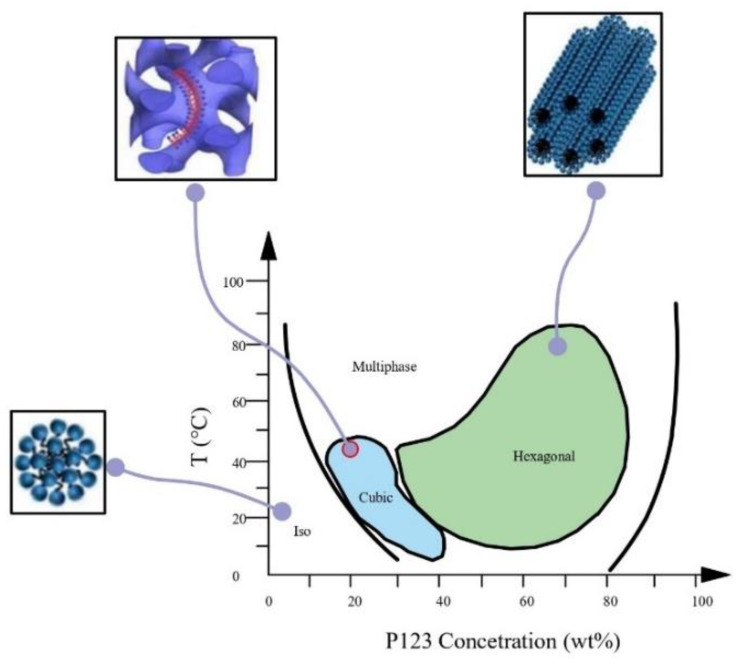
Phase diagram of the Pluronic P123 redrawn from [25].

Pluronic P123 (EO_20_PO_70_EO_20_), belonging to the PEO-PPO-PEO block copolymer series, has been widely used in cosmetics, pharmaceuticals, the food industry, etc. [26]. The phase behaviour in water was studied by Wanka et al. [25] and shown in Figure 1.

Different mesophases such as isotropic, cubic, and hexagonal are formed by increasing the copolymer concentration increases and changing the temperature. Based on this phase diagram, samples were made with three different concentrations of P123: 30 wt% (LLC30), 40 wt% (LLC40) and 45 wt% (LLC45). At these concentrations and in the temperature range 32–40 °C, the cubic and hexagonal phases in the P123/water binary system are reported. Typically, the hexagonal phase consists of rod-shaped micelles arranged in a three-dimensional hexagonal lattice [27]. While the cubic phase is characterised by a spherical arrangement, with the polar segment of the molecule positioned on the surface of the sphere and the non-polar segment residing in the centre of the sphere [28]. The incorporation of drugs into the mesophase composition could induce structural changes, so it was decided to test two hexagonal structures at 40% and 45%. The 40% P123/water hexagonal structure was identified as the system most affected by the introduction of a third component, as it is closest to the boundary phase between the cubic and hexagonal phases. Therefore, CA was added to P123, to assess its effect on the structural organisation of the LLC and the possible improvement of drug penetration into the skin. The amount of CA in the LLC was always 0.5 wt%. Visually, LLC 40 and LLC 45 had a gel-like consistency, high viscosity and birefringence, while LLC 30 was less viscous and isotropic.

**Figure 2:**
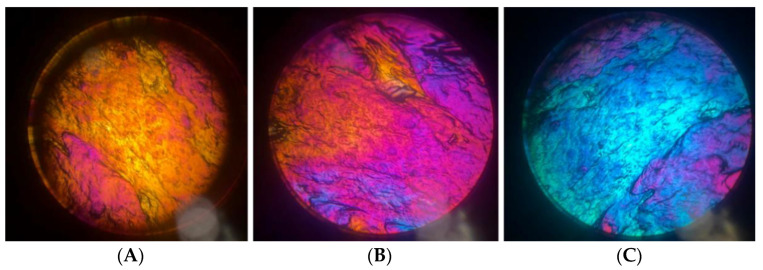
POM images of mesophase LLC45at (**A**) 25 °C, (**B**) 32 °C and (**C**) 40 °C.

The characterisation of liquid crystalline phases was carried out using the polarising optical microscope (POM). Indeed, hexagonal mesophases are anisotropic and birefringent as, having mostly two refractive indices, they can split the polarised light ray into two radiations that oscillate at different frequencies, while cubic mesophases are isotropic and they appeared as solutions. POM observation allowed us to identify hexagonal phases (LLC 40, LLC 45) because they are birefringent, but not the cubic phase (LLC 30), which is isotropic. Figure 2 shows the typical hexagonal texture of LLC 45 formulations at different temperature.

**Figure 3:**
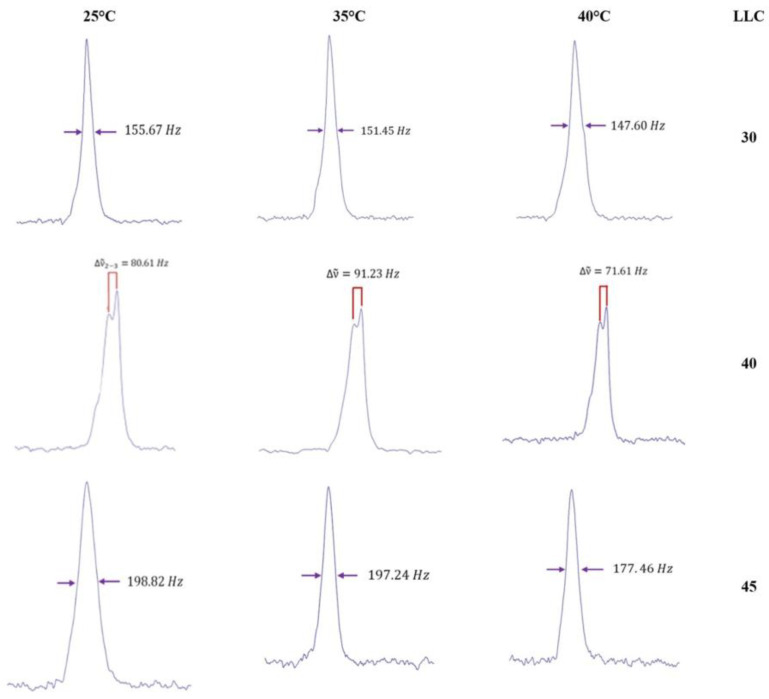
^2^H-NMR spectra of LLC 30, LLC 40 and LLC 45 at 25 °C, 35 °C and 40 °C.

**Figure 4:**
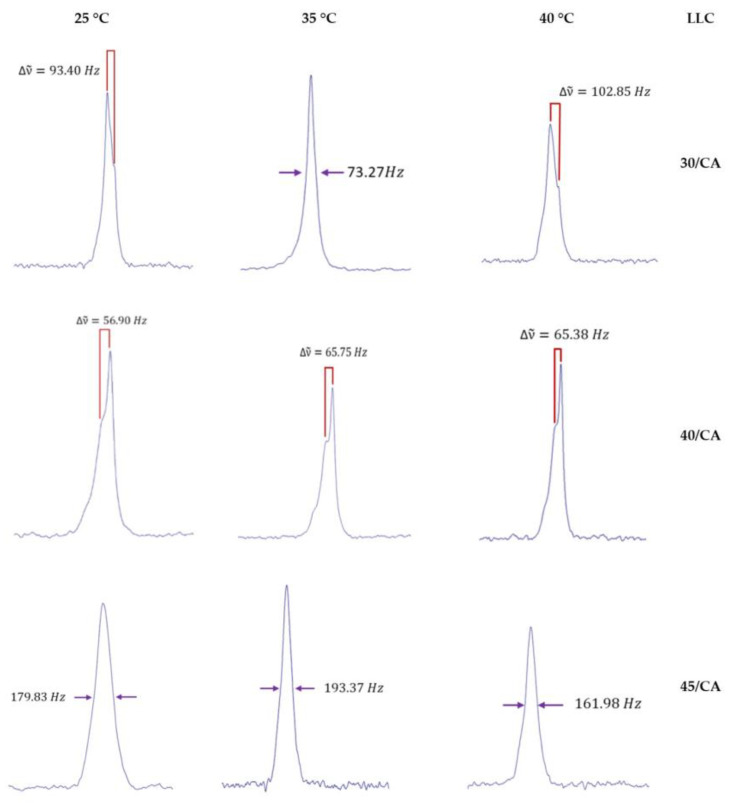
^2^H-NMR spectra of LLC 30/CA, LLC 40/CA and LLC 45/CA at 25 °C, 35 °C and 40 °C.

The LLC were analysed via magnetic nuclear resonance ^2^H-NMR. Empty LLC and CA-loaded LLC were evaluated at three temperatures (25 °C, 35 °C and 40 °C). LLC 30 showed a typical spectrum of cubic phase at each temperature, confirming the POM observation, while LLC 40 was shown as an indicative biaxial spectrum typical of hexagonal phase at each temperature; LLC 45 appears to show a uniaxial spectrum at all the recorded temperature, as shown in Figure 3, but the same formulation both under the microscope and observation in polarised light shows birefringence (Figure 2). This could be related to the lack of long-range order in the gel microstructure, or even to the fact that it takes a much longer time (as opposed to a few weeks) to reach homogeneity, as reported in the literature [29]. Therefore, the spectra obtained could be due to the presence of very small domains, which do not reveal the structure at NMR times. By introducing caffeic acid, even in a small percentage, disorder is generated in all structured phases as can be seen in Figure 4. The division of the quadrupolar peak or the width at half-height of the recorded spectrum becomes smaller as the drug is introduced.

### 2.2. Rheological Characterisation

For the steady flow experiments, the temperature and concentration dependence of the viscosity are reported in Table 1. Considering temperature over 30 °C, the data suggest the presence of a low-viscous gel at a lower concentration, while the system becomes more viscous with increase in the concentration of the P123 polymer. It is worth noting the fluidifying effect of the addition of CA in the systems marked by the decreasing of viscosity. The effect of the temperature is expected. The viscosity decreases with increasing temperature and this is due to kinetic effects. The LLC40/CA shows a higher viscosity value at 40 °C. These data suggest a structural modification along this isopletal line.

**Figure 5:**
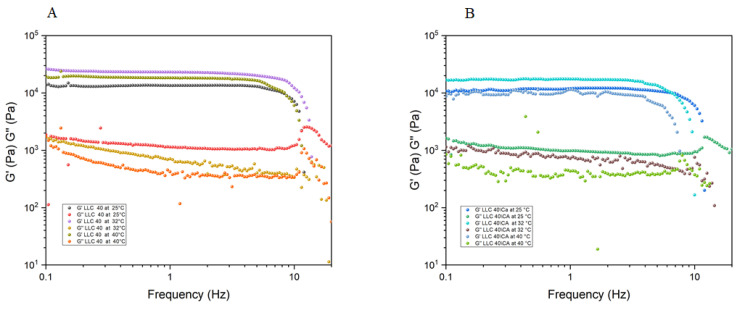
*G*’ and *G*” representation of (**A**) LLC 40 at 25 °C, 32 °C and 40 °C and (**B**) LLC 40/CA at 25 °C, 32 °C and 40 °C.

At a low temperature (25 °C), the viscosity is higher and at this temperature it is not possible to appreciate the effect of CA on the structure since the changes are within the experimental error. Typical strong gel spectra, consisting of two nearly horizontal straight lines on *G*′ and *G*″, were recorded. In Figure 5, the dynamic spectra for the 40% sample are shown. *G*′ is typically one order of magnitude greater than *G*″, quite apart from the temperature considered. However, it must be mentioned that while the mechanical profile for all mixtures appears similar, differences can be observed in the value of the elastic and viscous moduli. They are lower with CA addition and at higher temperatures.

**Figure 6:**
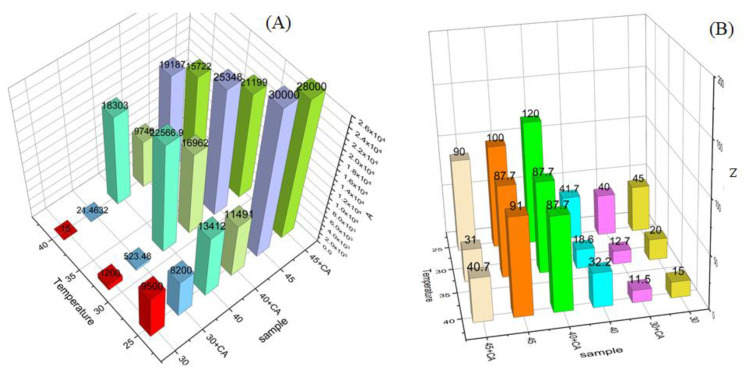
Weak-gel model parameters for the investigated formulations. The “gel strength”, *A* (**A**) and the “flow coordination number”, *z*, (**B**) are shown at different temperatures.

Dynamic shear experiments (Figure 6) show the composition and temperature dependence of the model parameters of the weak gel, *z* and *A*, obtained by fitting the viscoelastic data to Equation (5). The system exhibits a high flow coordination number, *z*, and high interaction strength values, *A*, in the hexagonal phase. Additionally, these data confirm the presence of the gel that becomes stronger with increasing polymer concentration. The flow coordination number jumps from a value close to 10 (cubic phase) up to about 90 for mixtures at 45 wt% when a hexagonal phase is formed. This means a hardening of the gel network and a different structural organisation in the hexagonal phase, characterised by the increase in *A* and *z*, respectively.

Once again, the effect of adding CA is evident for the LLC 30 and LLC 45 mixtures, where the *A* and *z* parameters decrease in presence of the CA and with the increasing of the temperature. The LLC 40/CA mixture, although weaker in terms of gel interactions (*A*), shows a higher coordination number than the mixture without CA at 32 °C; additionally, it keeps constant in temperature. This trend is reversed for all other investigated mixtures. The *A* and *z* decrease both with the addition of CA and with increasing temperature. It appears that CA induces a more coordinated tridimensional structure which is not modified by temperature.

### 2.3. In Vitro Diffusion and Ex Vivo Permeation Studies

**Figure 7:**
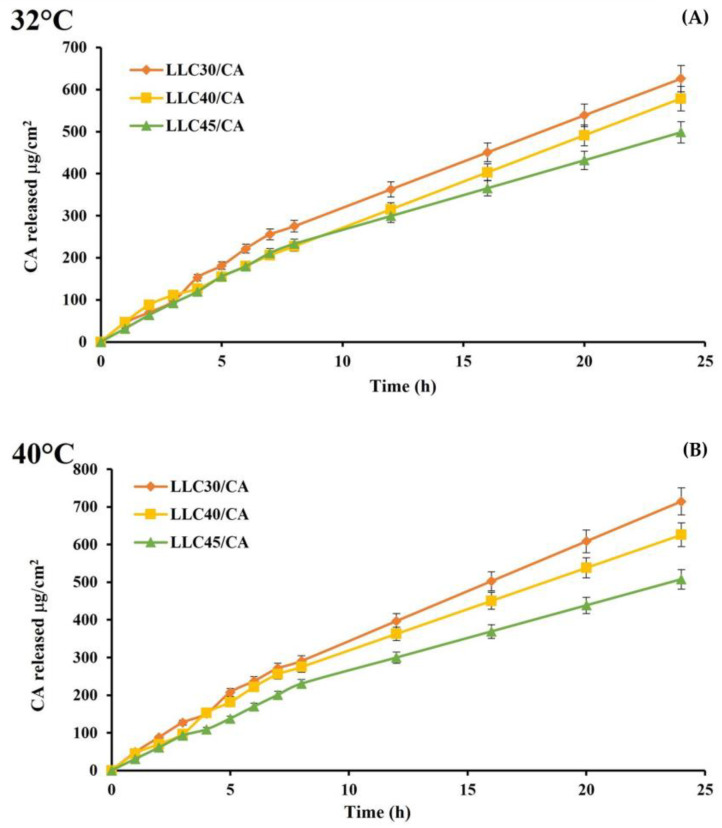
Diffusion profiles: (**A**) at 32 °C; (**B**) at 40 °C.

Diffusion studies (Figure 7) were performed using cellulose membranes for 24 h. LLC 30/CA was the formulation capable of releasing the highest amount of CA, corresponding to 625.67 μg/cm^2^ at 32 °C and 714.38 μg/cm^2^ at 40 °C. The other two structured phases showed a similar release profile at both temperatures.

The diffusion coefficient (*D*, cm^2^/s) for formulations investigated in diffusion studies at both 32 °C and 40 °C, were determined using Higuchi’s equation (Equation (1)) [30]:

(1) $$\frac{Q}{C_{0}}=2\;\sqrt{\frac{D}{\pi }}\sqrt{t}$$

where *Q* is the molar flow of the drug across the surface, *C*_0_ is the drug concentration and *t* is the release time.

The diffusion of drugs loaded in LLC phases is influenced by both structural factors and external factors such as temperature. Normally, the diffusion of drugs from cubic phases is faster than from hexagonal ones and the increase in temperature causes an increase in the diffusion of the loaded drug [31].

For our LLC formulations, diffusion coefficients (Table 2) were higher in cubic (LLC 30/CA) than hexagonal phase (LLC 40/CA and LLC 45/CA), while they were affected little by temperature in the LLC 40 and LLC45 formulations; in the LLC 30 formulation, the diffusion was more affected by increasing temperature, also shown by the rheological data.

The statistical analysis confirmed that by increasing temperature, a significant difference in drug release was observed (*p* value < 0.05) for LLC 30/CA, while LLC 40/CA and LLC 45/CA did not show no significant difference at temperatures tested.

**Figure 8:**
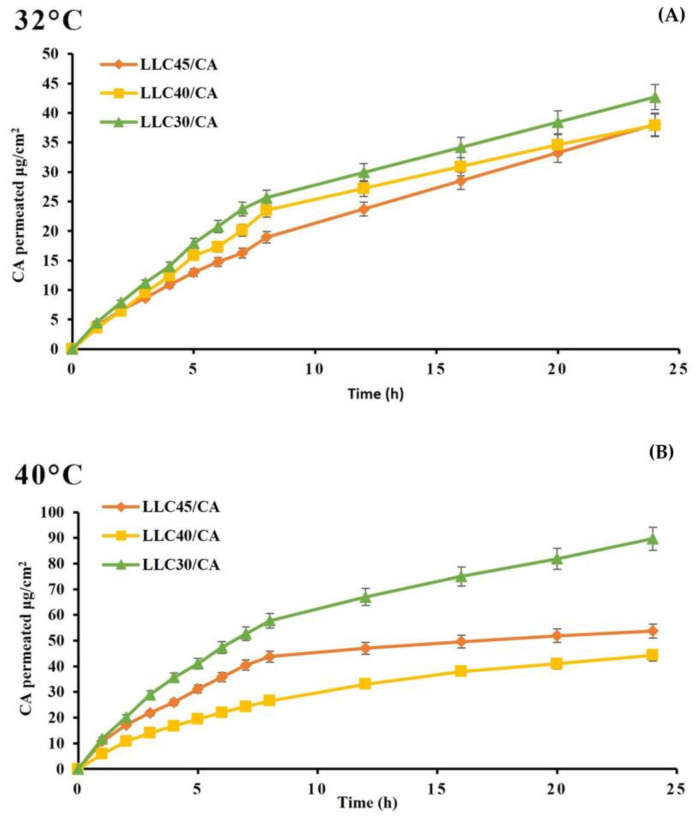
Permeation profiles through rabbit ear skin: (**A**) at 32 °C; (**B**) at 40 °C.

Permeation profiles of P123 lyotropic liquid crystals with CA were performed using rabbit ear skin, with vertical Franz diffusion cells for 24 h at 32 °C and 40 °C; the amount of released CA was assessed with UV spectroscopy at 286 nm. For LLCs, the release profile is strongly influenced by the phase taken up by microstructure, e.g., the cubic phase releases encapsulated drugs faster than the hexagonal phase [32,33]. At both temperatures, a faster and higher release can be seen for LLC 30/CA, 42.78 μg/cm^2,^ and 89.70 μg/cm^2^, respectively. Furthermore, at 40 °C, LLC 45/CA released faster than LLC 40/CA, in contrast to 32 °C (Figure 8).

In skin permeation studies, two parameters are crucial: the permeability coefficient (*Kp*) and the steady-state flux (*J_ss_*). The permeability coefficient is calculated from the flux and initial concentration (*Ci*) of polyphenols in the donor compartment, while the steady-state flux measures the amount of permeant crossing the membrane at a constant rate (Equation (2)) and is reported in Table 3:

(2) $$Kp=\frac{J_{ss}}{C_{i}}$$

All formulations increase the permeation rate of caffeic acid with increasing temperature, but only LLC 30/CA and LLC 45/CA double the amount of permeated drug. LLC 40/CA also has an anomalous behaviour: although the amount of permeated drug increases slightly, the flux and permeation coefficient decrease with increasing temperature.

### 2.4. Caffeic Acid Skin Retention Studies

Intracutaneous accumulation of the drug is a possibility to prolong its duration of action and improve drug treatment. For this reason, the amount retained in the skin after 24 h of release was evaluated. Results were compared at both temperatures and reported in Table 4. At 32 °C, the LLC45/CA formulation showed a higher accumulation in the skin 267.58 µg/cm^2^, but slower drug release. At 40 °C, in contrast, the LLC40/CA formulation showed a higher affinity for the skin layer (201.47 µg/cm^2^) than LLC45/CA (90.35 µg/cm^2^), but the amount of drug released over 24 h was comparable. These results confirm those obtained in the skin permeation study with LLC.

The data obtained seem to justify the reported flux and *Kp* values. In fact, the hexagonal structure of the LLC 40/CA sample presents a greater accumulation in the skin as the temperature increases, while the LLC 45/CA sample, although presenting the same hexagonal phase, reduces its accumulation in the skin by almost three times by increasing the amount permeated. The LLC 40/CA mixture appears to be affected in both diffusion and permeation by the increased coordination found in the dynamic rheology and viscosity studies.

LLC 30/CA showed a higher amount of drug permeation and a good CA retained in the skin, about 215 μg/cm^2^ and 169 μg/cm^2^, at different temperatures tested.

## 3. Conclusions

Caffeic acid loading in lyotropic liquid crystal based on the P123/water microstructures showed a good permeation profile and retention in the skin ensuring sustained drug release.

The addition of CA did not so much lead to changes in the LLC microstructure of binary system P123/water, as reported using the ^2^H-NMR spectra and POM observations, but rather modified its fluidity as showed the dynamic rheological measurements.

All experiments were carried out at 32 °C and 40 °C to reproduce the physiological and hyperthermic conditions typical of inflammatory-based diseases. The permeation profile of all formulations showed an increased steady-state flux (*J_ss_*), and a decreased skin drug retained at highest temperature tested.

Only LLC 40/CA formulation showed a different behaviour, in terms of NMR spectra, results of the rheology studies, and diffusion and permeation profiles, if compared to the similar hexagonal phase obtained through increasing the polymer concentration (LLC 45/CA). This may have been attributed to the greater coordination that caffeic acid establishes in this structure with the polymer chains, which determined an increase the CA skin retained at 40 °C.

The liquid crystalline mesophases obtained from Pluronic P123 could represent a good strategy for the skin treatment of a lipophilic drug such as caffeic acid.

In particular, the cubic phase LLC 30/CA shows a higher permeation profile and good accumulation in the skin at both temperatures tested.

## 4. Materials and Methods

Caffeic acid (CA), Pluronic 123 (P123), cellulose membranes and all solvents were purchased from Sigma Aldrich (Milan, Italy). Deionised water with 5 wt% deuterium oxide (Aldrich, Milan) was used in order to perform ^2^H-NMR measurements. Rabbit ear skin was provided by local farm that killed rabbits for slaughter purposes, where slaughter is defined as killing for human consumption. Therefore, these are waste materials from farms for which no ethical regulations are foreseen.

### 4.1. Liquid Lyotropic Crystals Gel Preparations

For preparations of Pluronic 123 LLC gels, a fixed drug percentage of CA (0.5% wt) and various ratios of Pluronic to water were used to obtain the different P123 lyotropic liquid phases [34]. Briefly, 25 mg of CA was mixed with an appropriate amount of water and block copolymer; details on sample preparations are given in Table 5. Subsequently, samples were centrifuged several times and stored at room temperature, or 4 °C, to ensure complete homogenisation. As some of samples exhibit negative thermoreological behaviour, they were liquid at low T, but LLC at room temperature [35]. Samples were evaluated and analysed after two weeks.

### 4.2. Optical Microscopy Observations

A Leica 12 Pol optical polarising microscope equipped with a heating unit was used for the phase characterisation of the samples. Indeed, the lyotropic liquid phases (except cubic phases) can be identified by comparing the typical textures of each liquid lyotropic phase with those reported in liquid crystal texture handbook [36].

### 4.3. ^2^H-NMR Theory and Experiments

**Figure 9:**
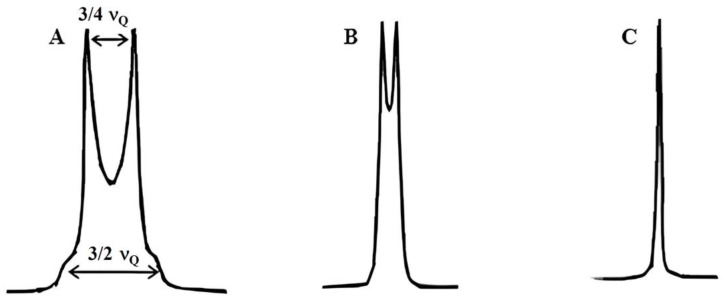
Typical ^2^H-NMR spectra of lamellar (**A**), hexagonal (**B**) and cubic (**C**) phase.

^2^H-NMR is employed in the examination of liquid crystals due to their manifestation of a distinctive NMR line contingent upon the anisotropy, geometry, and domain size of the liquid crystal aggregates, along with the motion of deuterated water molecules. When subjected to a magnetic field, liquid crystalline phases align in a manner influenced by the molecular diamagnetic susceptibility. ^2^H-NMR are predicated on quadrupolar interactions, determining the spectral frequency as follows (Equation (3)):

(3) $$\Delta \nu =\pm \frac{3}{8}\nu_{q}(3\;\cos^{2}\theta -\eta \;\sin^{2}\theta \cos2\varphi )$$

where ν_q_ is the partially averaged quadrupole coupling constant, θ and φ are the polar and azimuthal angles defining the direction of the external magnetic field in the aggregate frame and η is the asymmetry parameter. The theoretical ^2^H-NMR spectra, illustrated in Figure 9, exhibit distinct characteristics for different phases of a liquid lyotropic crystal: lamellar, hexagonal, and cubic. The lamellar structure displays a line shape characterised by two shoulders separated by a value of 3/2 ν_q,_ along with two edge singularities separated by a value of 3/4 ν_q_. The hexagonal phases exhibit a line shape like the lamellar phase but with a smaller separation between the shoulders and singularities. In the case of an isotropic sample, such as a cubic phase, a singlet is observed.

For the ^2^H-NMR experiments, a quadrupole echo sequence with a π/2 pulse width of 3.5 μs was employed. The delay between the two π/2 pulses was 40 μs and repetition was 1 s. Spectra were recorded 30 min after each temperature setting to allow samples to reach thermal equilibrium. The experiments were conducted using a Bruker AVANCE 300 pulsed superconducting spectrometer operating in Fourier Transform mode, at a resonance frequency of 46.53 MHz. The sample temperature was controlled by passing air through the sample holder at three different temperatures (25 ± 1 °C, 35 ± 1 °C and 40 ± 1 °C).

### 4.4. Rheological Characterisation

The rheological characterisation of P123 crystalline mesophases was conducted using a shear stress-controlled rheometer SR5000 (Rheometrics, Piscataway, NJ, USA) equipped with a plate-plate geometry (gap 1.00 mm, diameter 25). The temperature was controlled using a Peltier apparatus (±0.1 °C). All measurements were performed in triplicate. The error was calculated and it was around 3%. To prevent errors due to evaporation, measuring geometries were surrounded by a solvent trap containing water. Two different kinds of experiments were carried out: (a) steady flow experiments; (b) dynamic shear experiments, which were performed in a frequency range between 0.1 and 15.9 Hz. The small amplitude dynamic tests provided information on the linear viscoelastic behaviour of materials through the determination of the complex shear modulus (Equation (4)).

(4) $$G*\left(\omega \right)=G^{\prime }\left(\omega \right)+iG^{\prime \prime }\left(\omega \right)$$

where *G*′(ω) is the in phase (or storage) component and *G*″(ω) is the out-of-phase (or loss) component. G′(ω) is a measure of the reversible, elastic energy, while *G*″(ω) represents the irreversible viscous dissipation of the mechanical energy. The dependence of these quantities on the oscillating frequency gives rise to the so-called mechanical spectrum, allowing the quantitative rheological characterisation of studied materials. All dynamic rheological measurements were performed within the linear viscoelastic region. The Weak Gel Model [37] was also applied to oscillatory spectra (Equation (5)):

(5) $$\left|G*\left(\omega \right)\right|=\sqrt{G^{\prime }{\left(\omega \right)}^{2}+G^{\prime \prime }{\left(\omega \right)}^{2}}=A\omega^{\frac{1}{Z}}$$

where *A* is interpreted as the interaction strength between the rheological units, a sort of amplitude of cooperative interactions, and *z* as the coordination number, which corresponds to the number of flow units interacting with each other to give the observed flow response [38].

### 4.5. Percutaneous Permeation and Diffusion Release Studies

Permeation and diffusion release were performed using Franz’s vertical diffusion cells for 24 h at two different temperatures: 32 °C and 40 °C. For permeation studies, full-thickness rabbit ear skin from a local slaughterhouse was used, previously frozen at −18 °C and 2 h before the experiments pre-equilibrated in saline at room temperature. A portion of this skin was placed between the receptor and donor compartments, with the epidermal side exposed to environmental conditions and the dermal side facing the receptor solution. Portions of cellulose membranes (Spectra/Por^®^, cut-off 12–14 kDa) were used for the diffusion studies. For all formulated mesophases, 0.4 g of CA-LLC gel was loaded and covered with parafilm to prevent water loss. The receptor compartment was filled with 5.5 mL of distilled water. At regular intervals and up to 24 h, the medium was taken for analysis and filled with an equal volume of fresh water. The amount of drug in the samples was assessed using UV-VIS spectrometry. The experiments were repeated three times and expressed as mean ± SD.

### 4.6. Caffeic Acid Skin Retention Studies

To assess the amount of caffeic acid retained into the skin, after permeation studies, the skin was removed to Franz diffusion cells and placed in ethanol. The solution was filtered with 0.22 μm and evaluated using UV-Vis spectroscopy Millipore membrane filters and analysed. All experiments were conducted in triplicate and expressed as mean ± SD.

### 4.7. Statistical Analysis

All experiments were performed three times, and the results were expressed as mean ± SD. Statistical analysis was performed using a Student’s *t*-test and *p*-values of ≤0.05 were considered statistically significant.

## Figures and Tables

**Table 1:** Formulations viscosity values at 25 °C, 32 °C and 40 °C at 100 Pa of stress. Error percentage is about 3%.

Formulations	Eta Pa s(25 °C)	Eta Pa s(32 °C)	Eta Pa s(40 °C)
LLC 30	3 × 10^5^	0.3	0.25
LLC 30/CA	3 × 10^5^	0.2	0.18
LLC 40	8 × 10^5^	3 × 10^5^	2 × 10^5^
LLC 40/CA	8 × 10^5^	1 × 10^4^	2 × 10^5^
LLC 45	1 × 10^6^	1 × 10^6^	7 × 10^5^
LLC 45/CA	1 × 10^6^	4 × 10^5^	6 × 10^5^

**Table 2:** Diffusion coefficient (*D*)and R^2^ value of formulations tested. * *p* value < 0.05 32 °C vs. 40 °C.

T (°C)	LLC 30/CA	LLC 40/CA	LLC 45/CA
32 °C	*D* = 5.82 × 10^−15^ R^2^ = 0.9882	*D* = 3.86 × 10^−15^ R^2^ = 0.9881	*D* = 2.19 × 10^−15^ R^2^ = 0.9940
40 °C	* *D* = 7.38 × 10^−15^ R^2^ = 0.9817	*D* = 3.29 × 10^−15^ R^2^ = 0.9662	*D* = 2.29 × 10^−15^ R^2^ = 0.9869

**Table 3:** Permeation coefficient (*Kp*) and steady-state flux (*Jss*) values of formulations tested.

T (°C)	LLC 30	LLC 40	LLC 45
32 °C	*Jss* = 3.2196 *Kp* = 4.50 × 10^−4^	*Jss* = 3.0448 *Kp* = 3.65 × 10^−4^	*Jss* = 1.9829 *Kp* = 2.18 × 10^−4^
40 °C	*Jss* = 5.7526 *Kp* = 8.05 × 10^−4^	*Jss* = 2.516 *Kp* = 3.02 × 10^−4^	*Jss* = 4.7164 *Kp* = 5.19 × 10^−4^

**Table 4:** Amount of CA retained into skin at 32 °C and 40 °C for all formulations tested.

Formulations	CA Retained into Skin (µg/cm^2^) at 32 °C	CA Retained into Skin (µg/cm^2^) at 40 °C
LLC 30/CA	215.33	169.36
LLC 40/CA	163.38	201.47
LLC 45/CA	267.58	90.35

**Table 5:** LLC composition.

Formulation	P123 wt%	H_2_O wt%	CA wt%
LLC 30	30	70	-
LLC 40	40	60	-
LLC 45	45	55	-
LLC 30/CA	29.85	69.65	0.5
LLC 40/CA	39.80	59.70	0.5
LLC 45/CA	44.78	54.72	0.5

## Data Availability

The data presented in this study are available within this article.
